# “Eye and Ear Infirmary,” or Occulist and Aurist, by Way of Advertisement

**Published:** 1878-11

**Authors:** 


"Eye and Ear Infirmary," or "Oculist and Aurist," by way of Advertisement.—The Medical Society of the State of New York, at the annual meeting held June 18, 1878, passed the following resolution. The subject being presented by the legal proceedings recently instituted in the Niagara County Medical Societies:

"*Resolved*, That it is a breach of the Code of Medical Ethics of this Society for a member of a County Society, entitled to send delegates to this Society, to use upon signs, bill heads or advertisements, by way of advertising his profession, the words 'Eye and Ear Infirmary,' or 'Oculist and Aurist.'"

